# “What Matters” to Community-Dwelling Older Adults With Dementia and Their Family Caregivers: A Qualitative Pilot Study

**DOI:** 10.1177/30495334251358620

**Published:** 2025-07-23

**Authors:** Huey-Ming Tzeng, Yong-Fang Kuo, Monique R. Pappadis, Elizabeth A. Hennessy, Maribel M. Marquez-Bhojani, Samuel V. David, Demetress Harrell, Mukaila A. Raji

**Affiliations:** 1 The University of Texas Medical Branch, Galveston, TX, USA; 2 Hospice in the Pines, Lufkin, TX, USA

**Keywords:** Medicare, aging, dementia, mild neurocognitive disorders

## Abstract

Drawing on the Institute of Healthcare Improvement (IHI)’s 4Ms framework of an Age-Friendly Health System in primary care to incorporate “What Matters,” mentation, mobility, and medication, this qualitative/interview pilot study listened to “What Matters” most to community-dwelling Medicare beneficiaries with dementia and family caregivers in the context of Annual Wellness Visit (AWV). We then mapped identified “What Matters” themes (health goals and worries) to IHI-4Ms. Twenty-four caregivers and two patients participated in a one-time phone/Zoom interview. We observed three overarching goal themes (physical/mental health maintenance, cognitive maintenance, and independence) and six overarching worry themes (cognitive decline, behavioral changes, health decline leading to loss of independence, mobility issues, dehydration, and caregivers’ financial responsibility). The identified goals and worries were mapped to at least one of IHI-4Ms components, indicating the potential for addressing these through AWVs. Our study is the first to understand “What Matters” to Medicare beneficiaries with MCI/ADRD in the context of AWVs and to map the identified health goals and worries to IHI-4Ms. The valuable lessons learned in this pilot study underscore the potential need for practical health policy considerations to support dementia-friendly AWVs so that “What Matters” to patients with MCI/ADRD and caregivers can be heard/discussed during AWVs.

## Introduction

The Centers for Medicare and Medicaid Services (CMS) has offered free Annual Wellness Visits (AWVs) to U.S. Medicare beneficiaries aged 65 years and older since 2011 (Morgan et al., 2021). AWVs’ impact on care quality for those with mild cognitive impairment (MCI) or Alzheimer’s Disease and Related Dementias (ADRD) remains a critical policy and practice gap. Those patients/caregivers often rely on primary care services to address “What matters” most regarding care needs (e.g., priorities/needs important to patients with MCI/ADRD and disease experience (DiBenedetti et al., 2020; Hauber et al., 2023)). The Institute for Healthcare Improvement’s (IHI, 2022) advocates for incorporating its 4Ms framework of an Age-Friendly Health System into primary care practices: “What Matters,” mentation, mobility, and medication. The present study focuses on the connection between AWVs for patients with MCI/ADRD and IHI’s 4Ms framework in primary care practices.

AWVs include cognition and risk assessment, medication reconciliation, advance care planning consultation, and review and development of personalized prevention plans (Centers for Medicare and Medicaid Services Medicare Learning Network, 2025). AWVs were associated with reducing the risks of falls/fractures (Tzeng et al. (2022) and the timely detection of cognitive impairment (Tzeng et al. (2024). Optimal dementia care should reflect “What Matters” most to community-dwelling patients with MCI/ADRD and their family caregivers (Downer, Al Snih et al., 2021; Downer, Chou et al., 2021; Gozalo et al., 2011; Knox et al., 2021; Kuo et al., 2009; Liss et al., 2021; Mitchell, 2015; Nguyen et al., 2020; Shepard et al., 2021, 2023). Thus, AWVs could represent the ideal healthcare encounters for incorporating “What Matters” into wellness and preventive service conversations.

Bogardus et al. (1998) developed a taxonomy for classifying goals for patients with dementia. This taxonomy includes four axes: goal domain, specificity, timeframe, and challenge. They identified three overarching goal domains: functioning, psychosocial, and medical care matters. These researchers found that patients and family caregivers often require assistance in setting specific and achievable goals; the different goals for the same patient reflect the roles and skills that people bring to support the patient’s care and include disagreements among family caregivers of the same patient. Naik and Walling (2022) urged clinicians to document the goals of care conversations with patients (veterans without cognitive impairment) throughout the care journey to include health values (e.g., managing health concerning the balance between quality and quantity of life), objective and measurable health outcome goals, and care preferences to achieve outcome goals. Another study conducted by Simpson et al. (2021) explored factors contributing to the realization of health promotion recommendations that Medicare beneficiaries (without cognitive impairment) received during the most recent AWV. Seven health-promotion-realizing factors were identified: (1) preserving mental health, (2) stress (preventing beneficiaries from practicing health promotion recommendations), (3) ongoing social support, (4) environment-supporting physical activities, (5) recent abnormal lab/assessment values, (6) reminders of practicing health promotion behaviors, and (7) community resources to ease health care access barriers. However, they found that AWV documentation did not reflect the aforementioned factors for most participants. These researchers emphasized the need to personalize AWV recommendations and strategies to provide ongoing support to older adults between AWV visits.

Since no studies have used the 4Ms framework to bridge AWV components and “What Matters” to Medicare beneficiaries with MCI/ADRD and their family caregivers, three of our co-authors first mapped the required AWV components to IHI’s four 4Ms components and the six key actions related to the 4Ms framework that IHI urges ambulatory care settings to perform at least annually for each patient (IHI, 2022; Supplemental eTable S1). We found that AWV components failed to prioritize documenting “What Matters” to community-dwelling patients/caregivers as a gap in practice. “What Matters” (i.e., health-related goals or worries) interweaves with the other three 4Ms components and may require health maintenance/care interventions associated with mentation, mobility, and medication.

### Purpose of the Study

This descriptive qualitative interview pilot study aimed to identify the themes of “What Matters” most to their health (i.e., goals and worries) as reported by Medicare beneficiaries with MCI/ADRD and their family caregivers in the context of Medicare’s AWVs. We then mapped the identified “What Matters” themes (health goals and worries) from respondents to IHI’s mentation, mobility, and medication components related to the 4Ms framework of an Age-Friendly Health System (IHI, 2022).

## Methods

### Design and Data Source

We conducted this qualitative study using phone or Zoom video conferencing interviews to gather opinions from current family caregivers and patients with MCI/ADRD. We used convenience sampling to recruit participants into a one-time, one-on-one, semi-structured interview.

The University of Texas Medical Branch (UTMB) Institutional Review Board determined that this study met the federal regulations of a Quality Assessment/Quality Improvement project. All participants verbally agreed to participate in recorded interviews. We followed the Consolidated Criteria for Reporting Qualitative Research (COREQ) guidelines (Tong et al., 2007).

### Sample

The pilot study used convenience sampling of Medicare beneficiaries with MCI/ADRD and their family caregivers residing in Texas. We aimed to interview 25 participants; we completed 26 interviews. Family caregivers had to be ≥21 years old at the interview; currently caring for a person ≥65 years on Medicare with an MCI/ADRD diagnosis; and have attended at least one AWV within the past 12 months. Patient participants had to be independent enough to care for themselves, told by a healthcare provider they had MCI/ADRD, be ≥65 years, on Medicare, not living in a long-term nursing home, and have attended at least one AWV within the past 12 months. Participants must speak English or Spanish and be interviewed via telephone or videoconferencing/Zoom.

Recruitment efforts included posts on social media, emails, local community events, an adult day care program, one local geriatric clinic, and word of mouth; we did not do any cold calls. Two English-speaking nurse interviewers and one English-Spanish bilingual interviewer were trained by the first author and coached throughout the data collection period (November 2023‒September 2024) to ensure consistency across interviewers. Participants verbally agreed to participate during screening and again before the interview. Interviews lasted 20‒120 min (average = 30 min); we recorded the interviews. Post-interview, participants received a $25 gift card.

### Measures

We conducted interviews using a semi-structured topic guide based on the study purpose and IHI’s 4Ms framework (open and multi-choice questions, refined through community stakeholders’ insights). The guide included seven interview questions and 12 demographic characteristics (Supplemental eFigure S1 and eTable S2). The main interview questions analyzed in this paper were Q1 and Q2 (health-related goals and worries for the patient with MCI/ADRD).

### Analyses

The first author trained three interviewers and coached them throughout the data collection period to ensure consistency across interviewers. All interviews were transcribed manually by a third-party vendor in verbatim form. Two coders independently conducted initial descriptive inductive content analysis (using an inductive thematic coding process) of the first six transcriptions in Microsoft Word. Two coders discussed and agreed on the initial coding through consensus and applied the initial coding to the remaining transcripts. Both coders verified new codes and checked for duplicates (the agreement level was 97.2% between two coders for all open questions in the semi-structured interview guide; 98.4% for the responses to the interview questions analyzed in this present study). We used Microsoft Word in the coding process and IBM SPSS (IBM Corp, 2021) for organizing the identified themes. Then, two coders performed descriptive inductive content analysis on the identified codes to identify major themes using Microsoft Word. Two coders met via Zoom to compare their analyses and resolve any disagreements on the final themes. An interpretation meeting of the senior research team (three of the co-authors) refined and agreed upon the final themes. Then, the first author mapped the identified “What Matters” themes with IHI’s four 4Ms components and IHI’s six key actions. Another interpretation meeting of the senior research team (three of the co-authors) refined the mapping of the identified “What Matters” themes with IHI’s four 4Ms components and IHI’s six key actions. Descriptive analyses on the coded transcripts and themes were generated using IBM SPSS (IBM Corp, 2021).

## Results

Supplemental eTable S2, eTable S3, and eFigure S2 profile the 24 caregivers (18 female, 6 Hispanic, 12 White, 10 Black) and the two patients (both female, one Hispanic, one White).

### Themes for Health-Related Goals and Worries

We identified eight goals (Table 1 with selected quotes). The top three goal themes in frequencies were: (1) Stay healthy at home (*n* = 17, 65%); (2) Keep the patient as comfortable as possible with the highest quality of life (*n* = 8, 31%); and (3) Provide love and caring support (*n* = 8, 31%). We then grouped these eight goals into three overarching themes: physical and mental health maintenance, cognitive maintenance, and independence.

**Table 1.** “What Matters”: Family Caregiver Participants’ Health Goals and Worries for Patients With Dementia and the Patient’s Own Health Goals and Worries by Overarching Themes and Sample Quotes (*n* = 26, 24 Family Caregivers and 2 Patients).

Overarching themes of health goals	Sum of the interviews	Health goals identified from the interviews^a^	Selected quotes
Health goal A. Physical and mental health maintenance	Count = 38	A.1. Stay healthy at home (e.g., less anxiety, more daytime energy, fewer preventable incidents like falling). (*n* = 17)	One participant stated, “*My [the daughter of the patient] goals for her [the mother] are to prolong the progression of her dementia as long as we can.*” [#12] Another participant said, “*We hope that he [the patient, the father] stays at home and his dementia doesn’t get worse. We don’t want to move him into a nursing home.*” [#22]
		A.2. Keep the patient as comfortable as possible with the highest quality of life. (*n* = 8)	One participant expressed, “*For her [the patient] to live her life the best that she can in a situation where she could take care of herself to the best of her ability.*” [#18] Another participant stated, “*We want her [the patient] to be as physically healthy as possible. We recognize that she doesn’t have much of a life. If she got cancer, we would not go through radiation.*” [#4]
		A.3. Provide love and caring support. (*n* = 8)	One participant emphasized, “*Provide support for her [the patient, the mother] and let her know that she has someone who will care for her and love her as she has done for me [the caregiver, the son] growing up. I am trying to reciprocate the affection she’s shown me growing up.*” [#5]. Another participant shared, “*Get as much enjoyment out of the time she [the patient, the mother] has left. I have put in place caregiving support to help me as much as possible. So that I have more quality time with her.*” [#12]
		A.4. Keep the person with MCI/ADRD mobile and physically active. (*n* = 4)	One participant said, “*Mainly to keep him [the patient] active like he has been. He still goes to exercise and does some walking*.” [#3]
		A.5. Patient’s voice: “I just want to be happy.” (*n* = 1)	Captured in the theme.
Health goal B. Cognition maintenance	Count = 8	B.1. Delay cognition decline or slow the process. (*n* = 6)	Captured in the theme.
		B.2. Keep the mind of the person with MCI/ADRD active and engaged at home. (*n* = 2)	“*Our doctor (the same doctor for the patient and the family caregiver) looks at the whole family and then prescribes to help him [the patient] live a meaningful life.*” [#23]
Health goal C. Being independent	Count = 4	C.1. Keep patient out of group home or memory care. Keep the patient independent for as long as safely possible. (*n* = 4)	One participant said, “*Give her [the patient] the independence that she needs and is truly just entitled to.*” [#18]
Overarching themes of health worries	Sum of the interviews	Health worries identified from the interviews^a^	Selected quotes
Health worry A. Cognition decline	Count = 14	A.1. Cognition declines and may no longer recognize family caregivers. (*n* = 8)	“*Her [the patient’s] dementia will become severe, leading her to forget those important memories from before.*” [#9] “*I am concerned that it [the patient’s cognition decline] is going to get worse, and there is not a thing that I can do about it. I am concerned about where our life is going and how much togetherness we will have.*” [#3]
		A.2. Forgetting things and having difficulty understanding what doctors say. (*n* = 4)	“*It’s the constant reminders to her [the patient], sitting down and going through everything [*e.g., *doctor’s advice or treatment plan]. And then the next week, she doesn’t remember that conversation, and that’s so frustrating.*” [#12]
		A.3. Keep the patient from being taken advantage of [by people who might know what’s going on and try to capitalize on the patient’s cognitive impairment]. (*n* = 1)	Captured in the theme.
		A.4. Patient’s voice: Not remembering certain things and having problems with short-term memory. (*n* = 1)	Captured in the theme.
Overarching themes of health goals	Sum of the interviews	Health goals identified from the interviews^a^	Selected quotes
Health worry B. Behavioral changes	Count = 14	B.1. Getting irritated easily (e.g., not feeling safe, not being peaceful and happy). (*n* = 4)	“*My ability to keep her [the patient] calm is my biggest worry because I know when she gets stressed and upset, it takes its toll on her.*” [#18]. “*I don’t want her days to be where she [the patient] doesn’t know who we are, or where she doesn’t feel safe, or she’s afraid of us, or she doesn’t want to leave the house, or she tries to leave the house. I want her to be as happy and peaceful as possible.*” [#21]
		B.2. Wandering behaviors (e.g., at night and getting injured). (*n* = 4)	“*I had door chimes put on the doors to alert us when he [the patient] opens the door.*” [#3] “*My biggest fear is him [the patient] driving somewhere, getting lost, and being unsafe.*” [#14]
		B.3. Forgetting to eat and losing weight. (*n* = 3)	“*I [the daughter] don’t have the support during the daytime with her. I cannot find an adult daycare close to us. She stays in an apartment (a community) during the daytime while I go to work. I want to support her during the daytime because she forgets a lot, and she forgets to eat, and she’s lost weight. During the daytime, I have to send somebody up there because her brain tells her, "I’m full," and I’m like, "No, you’re not full; you haven’t eaten today." And so by me preparing one lunch at a time, when I go back to the apartment, and I look in the refrigerator, I see that she did not eat lunch because I didn’t have anybody to go over there and say, "it’s time for you to eat" and watch her eat. They may get her started, but when they shut the door, she feels like she’s full. So she has lost about 25* pounds. *I worry about eating and ensuring somebody is there [with my mother/the patient] during the daytime. If I can keep her functional, she’s walking around, she remembers long term, she doesn’t remember the short term, so if I can keep her functional as long as I can, then it allows me to work and be able to leave her with some monitoring system.*” [#10]
		B.4. Refusing help. (*n* = 3)	“*For sure, we get into many arguments [with the patient], and I have heard comments like: “I’m not brain dead yet,” and “I’ve taken care of my business my entire life plus grandmother.*” [#12]
Health worry C. Health decline leading to losing independence or moving to nursing homes	Count = 7	C.1. Health decline beyond the level family caregivers can manage. (*n* = 6)	“*Considering his [the patient’s] age, I’m fearful that we’ll be getting more and more sickness and with a new [illness] development sometime. It’s stressful at times, especially for him.*” [#7] “*We are worried about him [the patient] having to go into the nursing home one day. Something happening that may lead to him going to the nursing home or his health possibly getting worse.*” [#20]
		C.2. Patient’s voice: Physical health decline (e.g., related to prior health conditions and family history). (*n* = 1)	“*I have had 13 surgeries in my lifetime, and I know that anesthesia can cause memory issues, and I have had problems coming out of anesthesia when I have had major surgeries. As the years have progressed, especially these last two years, I have noticed it more, which is a big concern to me. My mother was diagnosed with Parkinson’s maybe about 3 years ago, and I don’t have Parkinson’s, but I know there are memory issues that relate to that. So, I don’t know if* that is *hereditary*.” [#6]
Health worry D. Mobility issues	Count = 3	D.1. Get injuries due to falling. (*n* = 2)	“*My biggest worry is when he [the patient] falls again, I can’t pick him up. He really didn’t get the proper training at rehab. He has been to rehab two* or *three times. The last time he was in was in June 2024 [within 3 months from the interview date]. He was there for 10 days, and he did quite well. And we haven’t had the kind of problems we had before, since they kept him that long [10 days, a longer time, at the post-acute rehabilitation facility].*” [#26]
		D.2. Not able to keep the patient moving [e.g., exercising]. (*n* = 1)	Captured in the theme.
Health worry E. Dehydration	Count = 2	E.1. Dehydration from dementia-related medications. (*n* = 2)	“*I am dealing with this whole Alzheimer’s thing; I have to really push fluids on her [the patient] because I guess all the medication she [the patient] is on really causing dehydration*.” [#17]
Health worry F. Family caregivers’ concerns related to financial responsibility	Count = 2	F.1. Family caregivers are physically constrained from care due to other/work commitments. (*n* = 1)	“*There are times that she [the patient] doesn’t remember things, and all she remembers is my name because it’s just the two of us. My dad died years back. It has not been easy for her, and now is the period in her life where she needs someone to be there for her. Someone that she recognizes and she can relate with*.” [#5]
		F.2. Insufficient financial resources and insurance for dementia care. (*n* = 1)	Same as the theme.

a Two coders independently conducted the initial content analysis using the first six transcriptions in Microsoft Word. To ensure accuracy, two coders met virtually to discuss any discrepancies until consensus was reached. Whenever either added new codes, the other coder verified the latest codes and checked for duplicates to ensure data rigor. Two coders reviewed the codes identified in one or two transcripts; with consensus, the selected codes were included in the further content analysis.

We identified 15 worries (Table 1 with selected quotes). The top 5 worry themes in frequencies were: (1) Cognition decline and may no longer recognize family caregivers (*n* = 8, 31%); (2) Health decline beyond the level family caregivers can manage (*n* = 6, 23%); (3) Forgetting things and having difficulty understanding what doctors say (*n* = 4, 15%); (4) Getting irritated easily (*n* = 4, 15%); and (5) Wandering behaviors (*n* = 4, 15%). We grouped these 15 worries into six overarching themes: cognition decline, behavioral changes, health decline leading to losing independence or moving to nursing homes, mobility issues, dehydration, and family caregivers’ worry related to financial responsibility.

### IHI Mapping With Identified Health-Related Goals and Worries

We mapped the eight identified goals (Q1; Supplemental eTable S4) to at least one of the IHI’s mentation, mobility, and medication components. The 15 identified worries (Q2; Supplemental eTable S4) were mapped to at least one of the IHI’s mentation, mobility, and medication components. Two worries were related to a family caregiver’s financial responsibility, and these two worries were mapped to IHI’s mentation component in Supplemental eTAble S4. Financial responsibility can impact access to or realization of health promotion recommendations related to patients’ mental health (mentation), mobility, and medication. Here, the mentation component in 4Ms is expanded also to include psychosocial issues, such as financial concerns.

## Discussion

This qualitative interview pilot study describes the themes of “What Matters” most to the health of Medicare beneficiaries with MCI/ADRD and their family caregivers. The identified “What Matters” themes (health goals and worries) from respondents were mapped to at least one of the IHI’s mentation, mobility, and medication components, suggesting the potential to be addressed through AWVs. Thus, many of the identified health goal/worry themes are not specific, realistic, or actionable; this observation could be due to a one-time interview using two open-ended interview questions to solicit insights via phone or Zoom (i.e., What are your goals for (name of the patient with dementia) health? What are your worries for (name of the patient with dementia) health?) as a study limitation. IHI (2022) recommended using previously validated questions or tools (e.g., functional status assessment) that are appropriate to patient factors (e.g., language and cultural background) to help patients and family caregivers express their health outcomes goals and care preferences. Naik and Walling (2022) emphasized that the goals of care conversations with patients (including advance care planning) are a progressive back-and-forth of health values, care preferences, and prognostic awareness, which may ultimately result in specific, realistic, and actionable outcome goals. The process of considering “What Matters” to patients from both their and their caregivers’ viewpoints is essential for primary care providers to align dementia care according to “What Matters” to each older adult with MCI/ADRD (Bogardus et al., 1998; IHI, 2022).

With only two patient participants with MCI/ADRD in this study, we were unable to draw meaningful conclusions from patients’ viewpoints. Similar to the findings of the study by Bogardus et al. (1998), the health goals expressed by the two persons with MCI/ADRD were non-specific. When family caregivers identified the goals and worries of persons with MCI/ADRD, the things labeled as “What Matters” most to the persons with MCI/ADRD appeared to be the things that mattered most to the family caregivers. Soliciting “What Matters” most to family caregivers of persons with MCI/ADRD is important and valuable, especially to support caregiving for community-dwelling persons with MCI/ADRD. “What Matters” most to the family caregiver is frequently misaligned with “What Matters” most to the patient with MCI/ADRD. Similarly, “What Matters” most to the clinicians or case managers is misaligned with “What Matters” most to the patient with MCI/ADRD (Bogardus et al., 1998).

In this study, with only two participants with MCI/ADRD, the overarching “What Matters” to patients with MCI/ADRD in the context of AWVs reflected the needs related to supporting caregivers at home and the emotional burden on caregivers. We learned that health goals centered on keeping patients comfortable at home and delaying the need to move to a nursing home. Health-related worries reflected the challenges of home-based caregiving and the grief about seeing loved ones with MCI/ADRD lose important memories, affection for family caregivers, emotional stability, and ability to care for themselves.

We observed that the health goals and worries identified in the present study (2 older adults with MCI/ADRD and 24 active family caregivers of older adults with MCI/ADRD) approximately mapped to the three broad goal domains (functional, psychosocial, and medical concerns) in the study (older adults with MCI/ADRD, family caregivers and healthcare providers) conducted by Bogardus et al. (1998), the health values in the study (older adults without MCI/ADRD) conducted by Naik and Walling (2022), and the health promotion-realizing factors (i.e., facilitators or barriers) identified in the study (older adults without MCI/ADRD) performed by Simpson et al. (2021). It appears that “What Matters” to patients, including health goals, worries, and health values, as well as facilitators and barriers to realizing wellness recommendations from AWVs, are similar terms that all lead to identifying practical directions for older adults with or without MCI/ADRD to live as fully as possible.

### Limitations

This pilot combined lessons learned from family caregivers and patients as appropriate. Information abstracted from the two patient interviews was limited, which is consistent with the study conducted by Bogardus et al. (1998). This study was small, with a sample of 26 Texas-based participants from a single geographic area. Thus, generalizability is limited, and conveying the findings to other contexts, study participants, or other clinical and non-clinical settings should be done with caution.

### Future Research Directions

The valuable information gained from this study suggests a practice gap in assisting older adults with MCI/ADRD and their caregivers in setting specific health goals and addressing concerns over their dementia care journey. This practice gap could lead to misalignment between care preferences related to health goals and worries and healthcare providers’ recommendations provided during AWVs and other maintenance visits. The lessons learned from mapping “What Matters” themes (health goals and worries) for patients with MCI/ADRD with IHI’s (IHI, 2022) mentation-mobility-medication components within the 4M framework of an Age-Friendly Health System underscore the potential need for practical health policy considerations to support dementia-friendly AWVs.

The findings of this Texas-based pilot study also will support a national family caregiver interview study using inductive content analysis for diverse populations. Future qualitative or mixed-methods studies should assess the role of social determinants of health and social identities of patients with MCI/ADRD and their family caregivers in “What Matters” most to their health during AWVs. In addition, future research should explore strategies (e.g., engaging the patient in expressing his or her activity preferences through showing pictures of gardening or baking) to identify “What Matters” most to the patient with MCI/ADRD and align the patient’s preferences with “What Matters” most to the family caregivers of the same patient. Eliciting “What Matters” most to family caregivers of the patients with MCI/ADRD is important and valuable as the dementia progresses. Healthcare providers should anticipate disagreements among family caregivers and resolve them with the best interest of the patient at heart.

### Conclusions and Policy Implications

Our study is the first to seek to understand “What Matters” to Medicare beneficiaries with MCI/ADRD or their caregivers in the context of AWVs and to map the identified “What Matters” themes (health goals and worries) with IHI’s mentation, mobility, and medication components related to the 4Ms framework of an Age-Friendly Health System (IHI, 2022). The identified “What Matters” themes were mapped to at least one of the IHI’s mentation, mobility, and medication components, indicating the potential for addressing these through AWVs. The valuable lessons learned in this study underscore the potential need for practical health policy considerations to support dementia-friendly AWVs so that “What Matters” to patients with MCI/ADRD and “What Matters” to their family caregivers can be heard and discussed during AWVs.

## Supplemental Material

sj-docx-1-ggm-10.1177_30495334251358620 – Supplemental material for “What Matters” to Community-Dwelling Older Adults With Dementia and Their Family Caregivers: A Qualitative Pilot Study Supplemental material, sj-docx-1-ggm-10.1177_30495334251358620 for “What Matters” to Community-Dwelling Older Adults With Dementia and Their Family Caregivers: A Qualitative Pilot Study by Huey-Ming Tzeng, Yong-Fang Kuo, Monique R. Pappadis, Elizabeth A. Hennessy, Maribel M. Marquez-Bhojani, Samuel V. David, Demetress Harrell and Mukaila A. Raji in Sage Open Aging
