# Supplementary material for: “What Matters” to Community-Dwelling Older Adults With Dementia and Their Family Caregivers: A Qualitative Pilot Study
Source: Sage Open Aging. 2025 Jul 23;11:30495334251358620. doi: 10.1177/30495334251358620 (PMC12290350; doi:10.1177/30495334251358620)
Supplement: sj-docx-1-ggm-10.1177_30495334251358620 – Supplemental material for “What Matters” to Community-Dwelling Older Adults With Dementia and Their Family Caregivers: A Qualitative Pilot Study [file sj-docx-1-ggm-10.1177_30495334251358620.docx]

Table of Contents for Supplementary eFigures and eTables

| Names | Page number(s) |
| --- | --- |
| eFigure S1. Interview questions. | pp. 2‒5 |
| eFigure S2. Participants’ locations and heat map. | pp. 6‒7 |
| eTable S1. Mapping the four components in the Institute of Healthcare Improvement’s (IHI) 4Ms framework of an age-friendly health system and IHI’s six key actions in ambulatory care settings with the required components for billing annual wellness visit (AWV) services. | pp. 8‒12 |
| **eTable S2.** Descriptive information of participant characteristics reported by family caregivers (n=24) and patient participants (n=2). | pp. 13‒16 |
| **eTable S3.** Brief profiles of the family caregiver and patient participants (n=26, 24 family caregivers and 2 patients). | p. 17 |
| **eTable S4.** Mapping the four components in the Institute of Healthcare Improvement’s (IHI) 4Ms framework of an age-friendly health system and IHI’s six key actions in ambulatory care settings with the required components for billing annual wellness visit (AWV) services. | pp. 18‒19 |

**eFigure S1.** Interview questions asked of both family caregivers and patient participants.

_________________________________________________________________________

A demographic question included in the introduction:

May I know your relationship with _______ (name of the patient with dementia)?

_01_ The patient’s spouse

_02_ The patient’s child

_03_ The patient’s brother

_04_ The patient’s sister

_05_ Other, please specify _______________

**Interview Questions:**

1. What are your goals for _ _____’s (name of the patient with dementia) health?

(What do you want to happen in the new few years related to patient’s health?)

2. What are your worries for _______’s (name of the patient with dementia) health?

2.a. __________ (name of the caregiver) What health and wellness services related to _______ (name of the loved one) are important to you?

(For patient interview only): _________ (name of the patient) What health, and wellness services are important to you?

2.b. Please share with me how to improve these important health and wellness services to _______ (name of the patient with dementia).

(For patient interview only): Please share with me how to improve these health and wellness services that are important to you.

3.a How often do you or another family member go to an Annual Wellness Visit with ___________ (name of the patient with dementia)?

(If needed, direct the caregiver to read the AWV infographic and repeat, "Annual Wellness Visits are free and there is no co-payment for people receiving Medicare. Annual Wellness Visits are wellness visits that happen once a year. Wellness visits cover wellness services like memory and fall risk assessment, medication questions, advanced care planning questions, and personalized prevention plans. This wellness visit includes a questionnaire that you complete ahead of the visit.")

_01_ Never (if the answer is never, please stop the interview.)

_02_ Sometimes _03_ Often _04_ Always

3.b How often do you or another family member go to an annual physical exam or illness doctor visit with ___________ (name of the patient with dementia)?

_01_ Never _02_ Sometimes _03_ Often _04_ Always

3.b. Comment: Please describe. _____________________

3.c Now, let us focus on your experience with the most recent wellness visit of _____ (name of the patient with dementia).

3.c.1 ____ (name of the caregiver), did the healthcare provider and clinic staff treat you and _______ (name of the patient with dementia) with respect? _00_ No _01_ Yes

3.c.1. Comment: Please describe.

___________________________________________________

3.c.2 Did the healthcare provider explain to you the purpose of a wellness visit?

_00_ No _01_ Yes

3.c.2. Comment: Please describe. __________________________

3.c.3 ______ (name of the caregiver), did the healthcare provider explain to you the risk assessment components included in a wellness visit? _00_ No _01_ Yes

3.c.3. Comment: Please describe. __________________________

3.c.4 What happened during the Annual Wellness Visit for _______ (name of the patient with dementia)?

_01_ Screening for memory loss or decline in daily activities

_02_ Timely referrals from the clinician

_03_ Assessment for risks (e.g., fall risk, home safety, depression)

_04_ Medication review and management

_04.1_ About the number of medications _______ (name of the patient with dementia) is taking

_04.2_ About what _______’s (name of the patient with dementia) medications are for

_04.3_ About what time to give _______ (name of the patient with dementia) medications

3.c.5 What parts of the Annual Wellness Visit are important to you for _______’s (name of the patient with dementia) health?

_01_ Screening for memory loss or decline in daily activities

_02_ Timely referrals from the clinician

_03_ Assessment for risks (e.g., fall risk, home safety, depression)

_04_ Medication review and management

_04.1_ About the number of medications _______ (name of the patient with dementia) is taking

_04.2_ About what _______’s (name of the patient with dementia) medications are for

_04.3_ About what time to give _______’s (name of the patient with dementia) medications

3.c.6 Did the healthcare provider explain to you the identified risks for ___’s (name of the patient with dementia) health and wellbeing? _00_ No _01_ Yes

3.c.6. Comment: Please describe. __________________________

3.c.7 _______ (name of the caregiver), did the healthcare provider explain what you could do to minimize the impact of these risks on _____’s (name of the patient with dementia) health and well-being? _00_ No _01_ Yes

3.c.7. Comment: Please describe. ___________________________

3.c.8 Were your questions about ______’s (name of the patient with dementia) health and well-being addressed? _00_ No _01_ Yes

3.c.8. Comment: Please describe. ___________________________

3.c.9 In general, did you find the wellness visits help you care for ____ (name of the patient with dementia)? _00_ No _01_ Yes

3.c.9. Comment: Please describe. ______________________________

1. _____ (name of the caregiver), please share with me how to improve the following wellness services for _______ (name of the patient with dementia).

4.a. Screening for memory loss or decline in daily activities

4.b. Timely referrals from the clinician

4.c. Assessment for risks that could impact _______’s (name of the patient with dementia) health and well-being (e.g., fall risk, home safety, depression)

4.d. Medication review and management

4.d.1 Review the medications _______ (name of the patient with dementia) is taking

4.d.2 Review the reasons for each of _______'s (name of the patient with dementia) medications for

4.d.3 Review the time to give each of _______’s (name of the patient with dementia) medications

1. Do you think the Medicare Annual Wellness Visit improves ______’s (name of the patient with dementia) quality of life? _00_ No _01_ Yes
2. Have you heard of the new FDA-approved dementia care medications (Leqembi/Lecanemab or Kisunla [Donanemab-azbt])? _00_ No _01_ Yes

6.a. Comment (if the response is Yes): Please share with me your thoughts: ________

1. Is _______ (name of the patient with dementia) able to express their opinion of their health care to you?

_00_ No _01_ Yes

7.a (If the response is Yes) What are _______’s (name of the patient with dementia) health goals?

Family caregiver’s words/statements: ________

Patient’s words/statements: ________

7.b (If the response is Yes) What are _______’s (name of the patient with dementia) health-related worries?

Family caregiver’s words/statements: ________

Patient’s words/statements: __________

**Demographics**

Your/Caregiver’s Sex: _00_ Female _01_ Male _02_ Unknown

How would you identify yourself/caregiver in terms of ethnicity?

_01_ Hispanic or Latino/a/x adult

_02_ Non-Hispanic or Non-Latino adult

_03_ Unknown (I prefer not to disclose) adult

How would you identify yourself/caregiver in terms of race? (choose all that apply):

_01_ White adult

_02_ Black adult

_03_ Asian adult

_04_ American Indian/Alaska Native adult

_05_ Hawaiian adult

_06_ More than one race

_07_ Unknown

_08_ Self describe: ___________

Your/caregiver’s age (in years): ___ Years old

Your/Caregiver’s Zip code and State of Residence (the primary residence):

Zip code: __________ State of Residence: ______________

Do you live with ______ (name of the patient with dementia)?

_00_ No _01_ Yes

If No, is _____ (name of the patient with dementia) living alone or with someone?

_01_ Alone

_02_ With relative(s) or the significant other

_03_ With friend(s)

_04_ Other, please describe: ___________

Sex of ______ (name of the patient with dementia): Patient’s Sex:

_00_ Female _01_ Male _02_ Unknown

How would you identify _______ (name of the patient with dementia) in terms of ethnicity?

_01_ Hispanic or Latino/a/x adult

_02_ Non-Hispanic or Non-Latino adult

_03_ Unknown (I prefer not to disclose)

How would you identify _______ (name of the patient with dementia) in terms of race? (choose all that apply):

_01_ White adult

_02_ Black adult

_03_ Asian adult

_04_ American Indian/Alaska Native adult

_05_ Hawaiian adult

_06_ More than one race

_07_ Unknown

_08_ Self describe: ___________

_______’s (name of the patient with dementia) age (in years): ______ years old

_______’s (name of the patient with dementia) zip code and State of Residence (the primary residence):

Zip code: __________ State of Residence: ______________

To the best of your knowledge, when were you first told that _______ (name of the patient with dementia) had dementia or was diagnosed with dementia?

___________ years ago

_________________________________________________________________________

**eFigure S2.** Participants’ locations and heat map.

eFigure S2.a. Participants’ locations were based on the provided zip codes.

| City | State | Zip Code | Country | Residential Area***** | Participants |
| --- | --- | --- | --- | --- | --- |
| Angleton | TX | 77515 | USA | Metropolitan | 1 |
| Austin | TX | 73301, 73344, 78652 | USA | Metropolitan | 3 |
| Baycliff | TX | 77518 | USA | Metropolitan | 1 |
| Bayou Vista | TX | 77563 | USA | Metropolitan | 1 |
| Dallas | TX | 75019 | USA | Metropolitan | 1 |
| Galveston | TX | 77550, 77551 | USA | Non-Metropolitan | 6 |
| Houston | TX | 77004, 77007, 77042, 77072, 77259 | USA | Metropolitan | 5 |
| League City | TX | 77573 | USA | Metropolitan | 2 |
| Pearland | TX | 77584 | USA | Metropolitan | 2 |
| Texas City | TX | 77590, 77591 | USA | Metropolitan | 2 |
| Tiki Island | TX | 77554 | USA | Non-Metropolitan | 2 |

* US Department of Agriculture Economic Research Service. Rural-Urban Continuum Codes. US Department of Agriculture Economic Research Service. Updated January 22, 2024. Accessed December 29, 2024. <https://www.ers.usda.gov/data-products/rural-urban-continuum-codes/>

eFigure S2.b. The heat map was based on the zip codes provided by the participants.

**
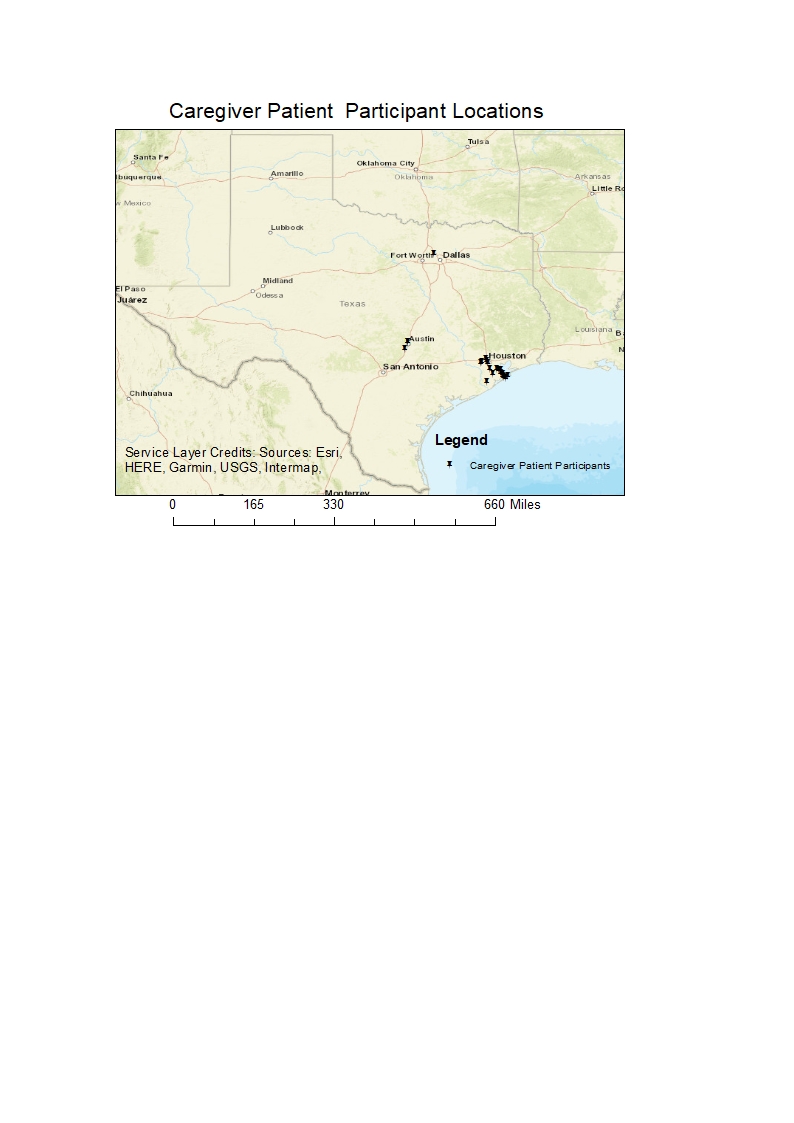
**

*Note.*

The zip code of each participant’s residence was used to create the heat map using ArcGIS software (*ArcGIS: Esri’s enterprise geospatial platform*. Esri Corp.; 2024. <https://www.esri.com/en-us/arcgis/geospatial-platform/overview>)

**eTable S1.**

*Mapping the four components in the Institute of Healthcare Improvement’s (IHI) 4Ms framework of an age-friendly health system* *and IHI’s six key actions in ambulatory care settings with the required components for billing annual wellness visit (AWV) services.*

| Medicare AWV required components | IHI's four components | | | | IHI's six key actions | | | | | |
| --- | --- | --- | --- | --- | --- | --- | --- | --- | --- | --- |
| AWV required components [The items below have been paraphrased for mapping purposes (e.g., including only one service component per item).]  yes = mapped  no = not mapped  pre = preparation tasks needed to deliver AWV services, which may apply to all IHI's four components in the IHI's 4Ms framework of an age-friendly health system and IHI's six key actions in ambulatory care settings. | What matters | Mentation | Mobility | Medication | Ask the older adult, "What Matters" | Document and align the care plan with “what matters” | Review, de-prescribe, dose-adjust, and avoid high-risk medications whenever possible | Screen for change in cognitive impairment and manage manifestations of cognitive impairment or refer for further specialist evaluation | Screen for depression, and, if positive, identify and manage contributors, initiate or refer for treatment | Screen for mobility limitations and address limitations to ensure safe mobility |
| 1. Prepare eligible patients for their Annual Wellness Visits by encouraging them to bring the following information to their appointment: 1) medical records, including immunization records; 2) detailed family health history; 3) full list of medications and supplements, including calcium and vitamins, and how often and how much of each they take; 4) full list of current providers and suppliers involved in their care, including community-based providers (for example, personal care, adult day care, and home-delivered meals), and behavioral health specialists. | no | pre | pre | pre | no | no | pre | pre | pre | pre |
| 2. The clinician or the patient updates the health risk assessment before or during the AWV. | no | pre | pre | no | no | no | no | pre | pre | pre |
| 3. Get patient self-reported information | no | pre | pre | no | no | no | no | pre | pre | pre |
| 4. Collect demographic data | no | pre | pre | no | no | no | no | pre | pre | pre |
| 5. Collect health status self-assessment | no | pre | pre | no | no | no | no | pre | pre | pre |
| 6. Identify the psychosocial risk of depression | no | yes | no | no | no | no | no | no | yes | no |
| 7. Identify the psychosocial risk of life satisfaction | no | yes | no | no | no | no | no | no | yes | no |
| 8. Identify the psychosocial risk of stress | no | yes | no | no | no | no | no | no | yes | no |
| 9. Identify the psychosocial risk of anger | no | yes | no | no | no | no | no | no | yes | no |
| AWV required components [The items below have been paraphrased for mapping purposes (e.g., including only one service component per item).]  yes = mapped  no = not mapped  pre = preparation tasks needed to deliver AWV services, which may apply to all IHI's four components in the IHI's 4Ms framework of an age-friendly health system and IHI's six key actions in ambulatory care settings. | What matters | Mentation | Mobility | Medication | Ask the older adult, "What Matters" | Document and align the care plan with “what matters” | Review, de-prescribe, dose-adjust, and avoid high-risk medications whenever possible | Screen for change in cognitive impairment and manage manifestations of cognitive impairment or refer for further specialist evaluation | Screen for depression and, if positive, identify and manage contributors, initiate or refer for treatment | Screen for mobility limitations and address limitations to ensure safe mobility |
| 10. Identify the psychosocial risk of loneliness or social isolation | no | yes | no | no | no | no | no | no | yes | no |
| 11. Identify the psychosocial risk of pain | no | yes | no | no | no | no | no | no | yes | no |
| 12. Identify the psychosocial risk of suicidality | no | yes | no | no | no | no | no | no | yes | no |
| 13. Identify the psychosocial risk of fatigue | no | yes | no | no | no | no | no | no | yes | no |
| 14. Identify the behavioral risk of tobacco use | no | no | no | no | no | no | no | no | no | no |
| 15. Identify the behavioral risk of low physical activity | no | no | no | no | no | no | no | no | no | no |
| 16. Identify the behavioral risk of poor nutrition | no | no | no | no | no | no | no | no | no | no |
| 17. Identify the behavioral risk of poor oral health | no | no | no | no | no | no | no | no | no | no |
| 18. Identify the behavioral risk of alcohol consumption | no | yes | no | no | no | no | no | no | yes | no |
| 19. Identify the behavioral risk of poor sexual health | no | yes | no | no | no | no | no | no | yes | no |
| 20. Identify the behavioral risk of lack of motor vehicle safety (e.g., seat belt use) | no | no | no | no | no | no | no | no | no | no |
| 21. Identify the behavioral risk of inadequate home safety | no | pre | pre | pre | no | no | pre | pre | pre | pre |
| 22. Assess ability to perform activities of daily living (ADLs), including dressing, feeding, toileting, and grooming, and physical ambulation, including balance or fall risks, and bathing. | no | pre | pre | pre | no | no | pre | pre | pre | pre |
| 23. Assess ability to perform instrumental ADLs (IADLs), including using the phone, housekeeping, laundry, transportation, shopping, managing medications, and handling finances. | no | pre | pre | pre | no | no | pre | pre | pre | pre |
| 24. Document updates to medical events of the patient's parents, siblings, and children, including hereditary conditions that place them at increased risk. | no | pre | pre | pre | no | no | pre | pre | pre | pre |
| 25. Document updates to past medical and surgical history (illnesses, hospital stays, operations, allergies, injuries, and treatments). | no | pre | pre | pre | no | no | pre | pre | pre | pre |
| 26. Document updates on the use of, or exposure to, medications, supplements, and other substances the person may be using. | no | no | no | yes | no | no | yes | no | no | no |
| 27. Update current patient providers and suppliers that regularly provide medical care, including those added because of the first and previous AWV personalized prevention plan services (PPPS). | no | pre | pre | pre | no | no | pre | pre | pre | pre |
| 28. Update current behavioral health providers | no | yes | no | no | no | no | no | no | yes | no |
| 29. Measure weight (or waist circumference, if appropriate) | no | pre | pre | pre | no | no | pre | pre | pre | pre |
| AWV required components [The items below have been paraphrased for mapping purposes (e.g., including only one service component per item).]  yes = mapped  no = not mapped  pre = preparation tasks needed to deliver AWV services, which may apply to all IHI's four components in the IHI's 4Ms framework of an age-friendly health system and IHI's six key actions in ambulatory care settings. | What matters | Mentation | Mobility | Medication | Ask the older adult, "What Matters" | Document and align the care plan with "what matters" | Review, de-prescribe, dose-adjust, and avoid high-risk medications whenever possible | Screen for change in cognitive impairment and manage manifestations of cognitive impairment or refer for further specialist evaluation | Screen for depression, and, if positive, identify and manage contributors, initiate or refer for treatment | Screen for mobility limitations and address limitations to ensure safe mobility |
| 30. Measure blood pressure | no | no | no | yes | no | no | yes | no | no | no |
| 31. Measure other appropriate routine measurements based on medical and family history. | no | pre | pre | pre | no | no | pre | pre | pre | pre |
| 32. Detect any cognitive impairments patients may have. | no | yes | no | no | no | no | no | yes | no | no |
| 33. Patients already have cognitive impairment symptoms. Clinicians may choose not to perform further testing to detect cognitive impairment changes. [Note: The authors added this item to provide clarification related to detecting any cognitive impairments patients may have for patients with an existing MCI/ADRD diagnosis.] | no | yes | no | no | no | no | no | yes | no | no |
| 34. When patients or family caregivers refuse to take cognitive assessment exams, clinicians may not perform further testing to detect cognitive impairment changes. [Note: The authors added this item to provide clarification related to detecting any cognitive impairments patients may have for patients with an existing MCI/ADRD diagnosis.] | yes | yes | no | no | yes | no | no | yes | no | no |
| 35. Assess cognitive function by direct or reported observations from the patient, family, friends, caregivers, and others. | no | yes | no | no | no | no | no | yes | no | no |
| 36. Identify possible factors contributing to increased cognitive impairment risk (e.g., using brief cognitive tests, factors related to health disparities and chronic conditions) | no | yes | no | no | no | no | no | yes | no | no |
| **37. Update the patient's written screening schedule** | no | yes | yes | no | no | no | no | yes | yes | yes |
| **38. The written screening schedule is based on the** recommendations of the United States Preventive Services Task Force and the CDC Advisory Committee on Immunization Practices (ACIP). | no | yes | yes | no | no | no | no | no | yes | yes |
| **39. Base written screening schedule on the** patient’s health risk assessment, health status and screening history, and age-appropriate preventive services Medicare covers. | no | yes | yes | no | no | no | no | yes | yes | yes |
| **40. Update the patient's list of risk factors and identified conditions.** | no | yes | yes | no | no | no | no | yes | yes | yes |
| 41. Update the list of mental health conditions, including depression, substance use disorders, and cognitive impairments. | no | yes | no | no | no | no | no | yes | yes | no |
| 42. Recommend primary, secondary, or tertiary interventions or report whether they are underway. | no | yes | yes | yes | no | no | yes | yes | yes | yes |
| 43. Update the list of treatment options and associated risks and benefits. | no | no | yes | yes | no | no | yes | yes | yes | yes |
| AWV required components [The items below have been paraphrased for mapping purposes (e.g., including only one service component per item).]  yes = mapped  no = not mapped  pre = preparation tasks needed to deliver AWV services, which may apply to all IHI's four components in the IHI's 4Ms framework of an age-friendly health system and IHI's six key actions in ambulatory care settings. | What matters | Mentation | Mobility | Medication | Ask the older adult, "What Matters" | Document and align the care plan with “what atters” | Review, de-prescribe, dose-adjust, and avoid high-risk medications whenever possible | Screen for change in cognitive impairment and manage manifestations of cognitive impairment or refer for further specialist evaluation | Screen for depression, and, if positive, identify and manage contributors, initiate or refer for treatment | Screen for mobility limitations and address limitations to ensure safe mobility |
| **44. As necessary, provide and update patient** AWV personalized prevention plan services (PPPS)**, including personalized health advice and appropriate referrals to health education or preventive counseling services or programs.** | no | yes | yes | no | no | yes | no | yes | yes | yes |
| 45. Make referrals to educational and counseling services or programs aimed at community-based lifestyle interventions to reduce health risks. | no | yes | yes | no | no | no | no | yes | yes | yes |
| 46. Make referrals to educational or counseling services or programs aimed at community-based lifestyle interventions to promote self-management and wellness (e.g., fall prevention, nutrition, physical activity, tobacco cessation, social engagement, weight loss, and cognition). | no | yes | yes | no | no | no | no | yes | yes | yes |
| **47. Provide advance care planning (ACP) services at the patient's discretion.** Medicare does not limit how often patients can revisit the ACP during the year, but cost sharing applies outside the AWV. | yes | no | no | no | yes | no | no | no | no | no |
| 48. Discuss advanced care planning (ACP) with the patient to prepare an advance directive if an injury or illness prevents them from making health care decisions. | yes | no | no | no | yes | no | no | no | no | no |
| 49. Discuss advance care planning (ACP) with the patient about future care decisions they might need or want to make. | yes | no | no | no | yes | no | no | no | no | no |
| 50. Discuss advanced care planning (ACP) with the patient to inform them how to let others know about their care preferences. | yes | no | no | no | yes | no | no | no | no | no |
| 51. Discuss advanced care planning (ACP) with the patient to identify their caregiver. | yes | no | no | no | yes | no | no | no | no | no |
| 52. Discuss advance care planning (ACP) with the patient about advance directive elements, which may involve completing standard forms. | yes | no | no | no | yes | no | no | no | no | no |
| **53. Review current opioid prescriptions.** [Note: Medicare now covers monthly chronic pain management and treatment services.] | no | no | no | yes | no | no | yes | no | no | no |
| 54. For a patient with a current opioid prescription: review any potential Opioid Use Disorder (OUD) risk factors, evaluate their pain severity and current treatment plan, provide information about non-opioid treatment options, and refer to a specialist, as appropriate. | no | yes | no | yes | no | no | yes | no | no | no |
| **55. Screen for potential substance use disorders (SUDs).** | no | yes | no | no | no | no | no | no | yes | no |
| 56. Review the patient's potential **substance use disorders (SUDs) r**isk factors, and as appropriate, refer them for treatment. Clinicians can use a screening tool, but it’s not required. The National Institute on Drug Abuse has screening and assessment tools. Implementing Drug and Alcohol Screening in Primary Care is a helpful resource. | no | yes | no | no | no | no | no | no | yes | no |
| 57. Perform a Social Determinants of Health (SDOH) Risk Assessment. [Note: Starting in 2024, Medicare includes an optional SDOH Risk Assessment as part of the AWV. This assessment must follow standardized, evidence-based practices, ensure communication aligns with the patient's educational, developmental, and health literacy level, and is culturally and linguistically appropriate.] | no | no | no | no | no | no | no | no | no | no |

*Notes.*

The key steps of mapping the AWV components to the 4Ms framework include: 1) the first author prepared the first version of the mapping; 2) the second coauthor and the last/senior coauthor carefully reviewed the mapping table and made the suggested changes; 3) the first author, the second coauthor and the last/senior coauthor discussed the preliminary mapping in a one-hour meeting and resolved the disagreements; 4) the first author presented the AWV-4Ms mapping table to the members of the community stakeholder advisory committee affiliated the parent research study; 4) the first author incorporated the suggested changes; 5) the first author sent the revised mapping table to the coauthors of this paper for a final review and approval.

ADRD = Alzheimer’s Disease and Related Dementias

AWV = annual wellness visit

IHI = The Institute of Healthcare Improvement’s (IHI)

IHI's 4Ms components: The IHI's 4Ms framework of an age-friendly health system includes the 4Ms components of "what matters," mentation, mobility, and medication.

IHI's six key actions = IHI underscored that six key actions in ambulatory care settings should occur at least annually or after a change in condition. These actions are to: 1) ask the older adult “what matters”; 2) document and align the care plan with “what matters”; 3) review, deprescribe, dose-adjust, and avoid high-risk medications whenever possible; 4) screen for change in cognitive impairment and manage manifestations of cognitive impairment or refer for further specialist evaluation; 5) screen for depression, and, if positive, identify and manage contributors, initiate or refer for treatment; and 6) screen for mobility limitations and address limitations to ensure safe mobility.

MCI = mild cognitive impairment

**eTable S2.**

*Descriptive information of participant characteristics reported by family caregivers (n=24) and patient participants (n=2).*

|  | **Family caregiver participants (n=24)** | | | **Persons with MCI/ADRD** | | |
| --- | --- | --- | --- | --- | --- | --- |
| Characteristics | N | % | Mean (Standard deviation [SD]) or comments | N | % | Mean (Standard deviation) or comments |
| **Family caregiver’s relationship with the patient with MCI/ADRD** |  |  |  |  |  |  |
| The patient’s spouse | 5 | 21 |  |  |  |  |
| The patient’s child | 16 | 67 |  |  |  |  |
| The patient’s brother | 0 | - |  |  |  |  |
| The patient’s sister | 0 | - |  |  |  |  |
| Other | 3 | 13 | Grandchild; mother-in-law; niece. |  |  |  |
| **Sex** |  |  |  |  |  |  |
| Female | 18 | 75 |  | 13 | 54 |  |
| Male | 6 | 25 |  | 11 | 46 |  |
| **Ethnicity** |  |  |  |  |  |  |
| Hispanic or Latino/a/x adult | 6 | 25 |  | 4 | 17 |  |
| Non-Hispanic or Non-Latino adult | 18 | 75 |  | 20 | 83 |  |
| **Race (choose all that apply)** |  |  |  |  |  |  |
| White adult | 12 | 50 |  | 13 | 54 |  |
| Black adult | 10 | 42 |  | 8 | 33 |  |
| Asian adult | 1 | 4 |  | 1 | 4 |  |
| American Indian or Alaska Native adult | 0 | - |  | 2 | 8 |  |
| More than one race | 2 | 8 |  | 1 | 4 |  |
| **Age (in years)** |  |  | Mean=51.25 (SD=16.80), range: 25‒82 |  |  | Mean=75.54 (SD=6.49), range: 65‒86 |

|  | | **Family caregiver participants (n=24)** | | | | | | **Persons with MCI/ADRD** | | | | | |  |
| --- | --- | --- | --- | --- | --- | --- | --- | --- | --- | --- | --- | --- | --- | --- |
| Characteristics | | N | | % | | Mean (Standard deviation [SD]) or comments | | N | | % | | Mean (Standard deviation) or comments | |  |
| **Residential area at the county level (converted from the zip code of the primary residence)** | |  | |  | |  | |  | |  | |  | |  |
| Metropolitan | | 16 | | 67 | |  | | 19 | | 79 | |  | |  |
| Non-Metropolitan | | 8 | | 33 | |  | | 5 | | 21 | |  | |  |
| **Living arrangement of the patient with MCI/ADRD** | |  | |  | |  | |  | |  | |  | |  |
| Living with the family caregiver | |  | |  | |  | | 17 | | 71 | |  | |  |
| Living alone | |  | |  | |  | | 3 | | 13 | |  | |  |
| Living with a relative(s) or the significant other | |  | |  | |  | | 3 | | 13 | |  | |  |
| Living with friend(s) | |  | |  | |  | | 0 | | - | |  | |  |
| Other | |  | |  | |  | | 1 | | 4 | | Living in an assisted living facility | |  |
| **Number of years since you were told that the patient had dementia or was diagnosed with dementia (in years)** | |  | |  | |  | |  | |  | |  | |  |
| ≤1 year ago | | 2 | | 8 | |  | |  | |  | |  | |  |
| >1 and ≤2 years ago | | 5 | | 21 | |  | |  | |  | |  | |  |
| >2 years and ≤3 years ago | | 3 | | 13 | |  | |  | |  | |  | |  |
| >3 years and ≤4 years ago | | 2 | | 8 | |  | |  | |  | |  | |  |
| >4 years and ≤5 years ago | | 4 | | 17 | |  | |  | |  | |  | |  |
| >5 years and ≤6 years ago | | 2 | | 8 | |  | |  | |  | |  | |  |
| >6 years and ≤7 years ago | | 3 | | 13 | |  | |  | |  | |  | |  |
| >7 years and ≤8 years ago | | 0 | | - | |  | |  | |  | |  | |  |
| >8 years and ≤9 years ago | | 0 | | - | |  | |  | |  | |  | |  |
| >9 years and ≤10 years ago | | 2 | | 8 | |  | |  | |  | |  | |  |
| >10 years ago | | 1 | | 4 | |  | |  | |  | |  | |  |
|  | | | **Patient participants with MCI/ADRD (they were their caregivers) (n=2)** | | | | | |  | | | | | |
| Characteristics | | | N | | % | | Mean (Standard deviation [SD]) or comments | |  | |  | |  | |
| **Sex** | | |  | |  | |  | |  | |  | |  | |
| Female | | | 2 | | 100 | |  | |  | |  | |  | |
| Male | | | 0 | | - | |  | |  | |  | |  | |
| **Ethnicity** | | |  | |  | |  | |  | |  | |  | |
| Hispanic or Latino/a/x adult | | | 1 | | 50 | |  | |  | |  | |  | |
| Non-Hispanic or Non-Latino adult | | | 1 | | 50 | |  | |  | |  | |  | |
| **Race (choose all that apply)** | | |  | |  | |  | |  | |  | |  | |
| White adult | | | 1 | | 50 | |  | |  | |  | |  | |
| Black adult | | | 1 | | 50 | |  | |  | |  | |  | |
| Asian adult | | | 0 | | - | |  | |  | |  | |  | |
| American Indian or Alaska Native adult | | | 0 | | - | |  | |  | |  | |  | |
| More than one race | | | 0 | | - | |  | |  | |  | |  | |
| **Age (in years)** | | |  | |  | | Mean=68.50 (SD=7.78), range: 63‒74 | |  | |  | |  | |
| **Residential area at the county level (converted from the zip code of the primary residence)** | | |  | |  | |  | |  | |  | |  | |
| Metropolitan | | | 2 | | 100 | |  | |  | |  | |  | |
| Non-Metropolitan | | | 0 | | - | |  | |  | |  | |  | |
| **Living arrangement of the patient with MCI/ADRD** | | |  | |  | |  | |  | |  | |  | |
| Living with the family caregiver | | | 0 | | - | |  | |  | |  | |  | |
| Living alone | | | 0 | | - | |  | |  | |  | |  | |
| Living with a relative(s) or the significant other | | | 2 | | 100 | |  | |  | |  | |  | |
| Living with friend(s) | | | 0 | | - | |  | |  | |  | |  | |
| Other | | | 0 | | - | |  | |  | |  | |  | |
|  | | | **Patient participants with MCI/ADRD (they were their caregivers) (n=2)** | | | | | |  | | | | | |
| Characteristics | | | N | | % | | Mean (Standard deviation [SD]) or comments | |  | |  | |  | |
| **Number of years since you were told that you had dementia or was diagnosed with dementia (in years)** | | |  | |  | |  | |  | |  | |  | |
| ≤1 year ago | | | 0 | | - | |  | |  | |  | |  | |
| >1 and ≤2 years ago | | | 0 | | - | |  | |  | |  | |  | |
| >2 years and ≤3 years ago | | | 2 | | 100 | |  | |  | |  | |  | |
| >3 years and ≤4 years ago | | | 0 | | - | |  | |  | |  | |  | |
| >4 years and ≤5 years ago | | | 0 | | - | |  | |  | |  | |  | |
| >5 years and ≤6 years ago | | | 0 | | - | |  | |  | |  | |  | |
| >6 years and ≤7 years ago | | | 0 | | - | |  | |  | |  | |  | |
| >7 years and ≤8 years ago | | | 0 | | - | |  | |  | |  | |  | |
| >8 years and ≤9 years ago | | | 0 | | - | |  | |  | |  | |  | |
| >9 years and ≤10 years ago | | | 0 | | - | |  | |  | |  | |  | |
| >10 years ago | | | 0 | | - | |  | |  | |  | |  | |

ADRD = Alzheimer’s Disease and Related Dementias

MCI = mild cognitive impairment

**eTable S3.**

*Brief profiles of the family caregiver and patient participants (n=26, 24 family caregivers and 2 patients).*

| Participant ID and role as a family caregiver (abbreviated as FC) vs. patient | Sex | Ethnicity: Hispanic or Latino/a/x (Hispanic) vs. Non-Hispanic or non-Latino (non-Hispanic) | Race | Age | Residential area at the county level* |
| --- | --- | --- | --- | --- | --- |
| 1 (FC) | Male | Non-Hispanic adult | White adult | 82 | Metropolitan |
| 2 (FC) | Male | Non-Hispanic adult | Asian adult | 45 | Non-Metropolitan |
| 3 (FC) | Female | Non-Hispanic adult | White adult | 80 | Metropolitan |
| 4 (FC) | Female | Non-Hispanic adult | White adult | 63 | Non-Metropolitan |
| 5 (FC) | Male | Non-Hispanic adult | Black adult | 25 | Metropolitan |
| 6 (Patient) | Female | Hispanic adult | White adult | 63 | Metropolitan |
| 7 (FC) | Male | Hispanic adult | Black adult | 31 | Metropolitan |
| 8 (FC) | Female | Non-Hispanic adult | White adult | 42 | Non-Metropolitan |
| 9 (FC) | Male | Hispanic adult | Black adult | 31 | Metropolitan |
| 10 (FC) | Female | Non-Hispanic adult | Black adult | 51 | Metropolitan |
| 11 (Patient) | Female | Non-Hispanic adult | Black adult | 74 | Metropolitan |
| 12 (FC) | Female | Non-Hispanic adult | White adult | 53 | Metropolitan |
| 13 (FC) | Female | Non-Hispanic adult | White adult | 79 | Metropolitan |
| 14 (FC) | Female | Non-Hispanic adult | White adult | 50 | Non-Metropolitan |
| 15 (FC) | Male | Non-Hispanic adult | Black adult | 28 | Metropolitan |
| 16 (FC) | Female | Non-Hispanic adult | Black adult | 31 | Metropolitan |
| 17 (FC) | Female | Hispanic adult | White adult | 61 | Non-Metropolitan |
| 18 (FC) | Female | Non-Hispanic adult | White adult | 45 | Metropolitan |
| 19 (FC) | Female | Non-Hispanic adult | Black adult | 52 | Non-Metropolitan |
| 20 (FC) | Female | Hispanic adult | Black adult | 46 | Metropolitan |
| 21 (FC) | Female | Non-Hispanic adult | Black adult | 44 | Metropolitan |
| 22 (FC) | Female | Hispanic adult | Black adult | 45 | Metropolitan |
| 23 (FC) | Female | Non-Hispanic adult | White adult | 70 | Metropolitan |
| 24 (FC) | Female | Non-Hispanic adult | White adult, more than one race | 51 | Non-Metropolitan |
| 25 (FC) | Female | Hispanic adult | More than one race | 49 | Metropolitan |
| 26 (FC) | Female | Non-Hispanic adult | White adult | 76 | Non-Metropolitan |

* US Department of Agriculture Economic Research Service. Rural-Urban Continuum Codes. US Department of Agriculture Economic Research Service. Updated January 22, 2024. Accessed December 29, 2024. <https://www.ers.usda.gov/data-products/rural-urban-continuum-codes/>

**eTable S4.**

“What Matters”: Mapping the components in the Institute of Healthcare Improvement’s (IHI) 4Ms framework of an age-friendly health system and IHI’s key actions in ambulatory care settings with family caregiver participants’ health goals and worries for patients with dementia and the patient’s own health goals and worries.

| Themes |  | IHI's four components | | | | IHI's six key actions | | | | | |
| --- | --- | --- | --- | --- | --- | --- | --- | --- | --- | --- | --- |
| Yes, AWVs may address the health goal = mapped  No, AWVs may not address the health goal = not mapped  “-” = not applicable  **Themes of “What Matters”: Health goals for the patients with dementia identified from the interviews** | Frequency among 26 participants (percent) | **What matters** | Mentation | Mobility | Medication | Ask the older adult, "what matters." | Document and align the care plan with “what matters” | Review, de-prescribe, dose-adjust, and avoid high-risk medications whenever possible | Screen for change in cognitive impairment and manage manifestations of cognitive impairment or refer for further specialist evaluation | Screen for depression, and, if positive, identify and manage contributors, initiate or refer for treatment | Screen for mobility limitations and address limitations to ensure safe mobility |
| 1. Stay healthy at home (e.g., less anxiety, more daytime energy, fewer preventable incidents like falling). | 17  (65) | yes | yes | yes | yes | - | - | yes | no | yes | yes |
| 2. Keep the patient as comfortable as possible with the highest quality of life. | 8  (31) | yes | yes | yes | yes | - | - | yes | no | yes | yes |
| 3. Provide love and caring support. | 8  (31) | yes | yes | no | no | - | - | no | no | yes | no |
| 4. Delay cognition decline or slow the process. | 6  (23) | yes | yes | no | no | - | - | no | yes | No | no |
| 5. Keep the person with MCI/ADRD mobile and physically active. | 4  (15) | yes | no | yes | no | - | - | no | no | No | yes |
| 6. Keep patient out of group home or memory care. Keep the patient independent for as long as safely possible. | 4  (15) | yes | yes | yes | no | - | - | no | yes | yes | yes |
| 7. Keep the mind of the person with MCI/ADRD active and engaged at home. | 2  (8) | yes | yes | no | no | - | - | no | no | yes | no |
| 8. Patient’s voice: “I just want to be happy.” | 1  (4) | yes | yes | no | no | - | - | no | no | yes | no |
| **Themes of “What Matters”: Health worries for the patients with dementia identified from the interviews** |  | IHI's four components | | | | IHI's six key actions | | | | | |
| 1. Cognition declines and may no longer recognize family caregivers. | 8  (31) | yes | yes | no | no | - | - | no | yes | No | no |
| 2. Health declines beyond the level family caregivers can manage. | 6  (23) | yes | yes | yes | no | - | - | no | yes | yes | yes |
| 3. Forgetting things and having difficulty understanding what doctors say. | 4  (15) | yes | yes | no | no | - | - | no | yes | No | no |
| 4. Getting irritated easily (e.g., not feeling safe, not being peaceful and happy). | 4  (15) | yes | yes | no | no | - | - | no | no | yes | no |
| 5. Wandering behaviors (e.g., at night and getting injured). | 4  (15) | yes | yes | no | no | - | - | no | yes | No | no |
| 6. Forgetting to eat and losing weight. | 3  (12) | yes | yes | no | no | - | - | no | yes | No | no |
| 7. Refusing help. | 3  (12) | yes | yes | no | no | - | - | no | yes | No | no |
| 8. Dehydration from dementia-related medications. | 2  (8) | yes | no | no | yes | - | - | yes | no | No | no |
| 9. Get injuries due to falling. | 2  (8) | yes | no | yes | no | - | - | no | no | No | yes |
| 10. Patient’s voice: Physical health decline. | 1  (4) | yes | no | yes | no | - | - | no | no | No | yes |
| 11. Not able to keep the patient moving. | 1  (4) | yes | no | yes | no | - | - | no | no | No | yes |
| 12. Keep the patient from being taken advantage of. | 1  (4) | yes | yes | no | no | - | - | no | yes | No | no |
| 13. Patient’s voice: Not remembering certain things and having problems with short-term memory. | 1  (4) | yes | yes | no | no | - | - | no | yes | No | no |
| 14. Family caregivers are physically constrained from care due to other/work commitments. | 1  (4) | yes | yes | no | no | - | - | no | no | yes | no |
| 15. Insufficient financial resources and insurance for dementia care. | 1  (4) | yes | yes | no | no | - | - | no | no | yes | no |

*Notes:*

* Two coders independently conducted the initial content analysis using the first six transcriptions in Microsoft Word. To ensure accuracy, two coders met virtually to discuss any discrepancies until consensus was reached. Whenever either added new codes, the other coder verified the latest codes and checked for duplicates to ensure data rigor. Two coders reviewed the codes identified in one or two transcripts; with consensus, the selected codes were included in the further content analysis.

4Ms components: The IHI’s 4Ms framework of an age-friendly health system includes the 4Ms components of “what matters,” mentation, mobility, and medication.

AWV = annual wellness visit

Health goals for the patient with MCI/ADRD: These goals were abstracted from the interview transcriptions for the question: What are your goals for _____’s (name of the patient with dementia) health? A participant may express no or more than one health goal.

Health worries for the patient with MCI/ADRD: These goals were abstracted from the interview transcriptions for the question: What are your worries for _______’s (name of the patient with dementia) health? A participant may express no or more than one health worry.

IHI = The Institute of Healthcare Improvement’s (IHI)

IHI's six key actions = IHI underscored that six key actions in ambulatory care settings should occur at least annually or after a change in condition. These actions are to: 1) ask the older adult “what matters”; 2) document and align the care plan with “what matters”; 3) review, deprescribe, dose-adjust, and avoid high-risk medications whenever possible; 4) screen for change in cognitive impairment and manage manifestations of cognitive impairment or refer for further specialist evaluation; 5) screen for depression, and, if positive, identify and manage contributors, initiate or refer for treatment; and 6) screen for mobility limitations and address limitations to ensure safe mobility.
